# Research progress of SREBP and its role in the pathogenesis of autoimmune rheumatic diseases

**DOI:** 10.3389/fimmu.2024.1398921

**Published:** 2024-08-19

**Authors:** Xiaofen Xu, Wumeng Jin, Runyu Chang, Xinghong Ding

**Affiliations:** Key Laboratory of Chinese Medicine Rheumatology of Zhejiang Province, School of Basic Medical Sciences, Zhejiang Chinese Medical University, Hangzhou, China

**Keywords:** sterol regulatory element binding proteins, cytokine storm, autoantibodies, autoimmune rheumatic diseases, inflammation, immune cells

## Abstract

Autoimmune rheumatic diseases comprise a group of immune-related disorders characterized by non-organ-specific inflammation. These diseases include systemic lupus erythematosus (SLE), rheumatoid arthritis (RA), ankylosing spondylitis (AS), gout, among others. Typically involving the hematologic system, these diseases may also affect multiple organs and systems. The pathogenesis of autoimmune rheumatic immune diseases is complex, with diverse etiologies, all associated with immune dysfunction. The current treatment options for this type of disease are relatively limited and come with certain side effects. Therefore, the urgent challenge remains to identify novel therapeutic targets for these diseases. Sterol regulatory element-binding proteins (SREBPs) are basic helix-loop-helix-leucine zipper transcription factors that regulate the expression of genes involved in lipid and cholesterol biosynthesis. The expression and transcriptional activity of SREBPs can be modulated by extracellular stimuli such as polyunsaturated fatty acids, amino acids, glucose, and energy pathways including AKT-mTORC and AMP-activated protein kinase (AMPK). Studies have shown that SREBPs play roles in regulating lipid metabolism, cytokine production, inflammation, and the proliferation of germinal center B (GCB) cells. These functions are significant in the pathogenesis of rheumatic and immune diseases (Graphical abstract). Therefore, this paper reviews the potential mechanisms of SREBPs in the development of SLE, RA, and gout, based on an exploration of their functions.

## Graphical Abstract

Under the influence of factors such as PUFAs and LXR, SREBP mRNA transcription is promoted, allowing the SCAP-SREBP complex to move from the endoplasmic reticulum to the Golgi apparatus. After cleavage by S1P and S2P in the Golgi, the complex enters the nucleus. Furthermore, under the influence of amino acids and the PI3K/AKT/mTOR signaling pathway, nuclear SREBP promotes the expression of target genes, thereby facilitating lipid synthesis, cytokine production, and accelerating inflammatory and immune cell responses. These functions are closely associated with the pathogenesis of rheumatic autoimmune diseases.

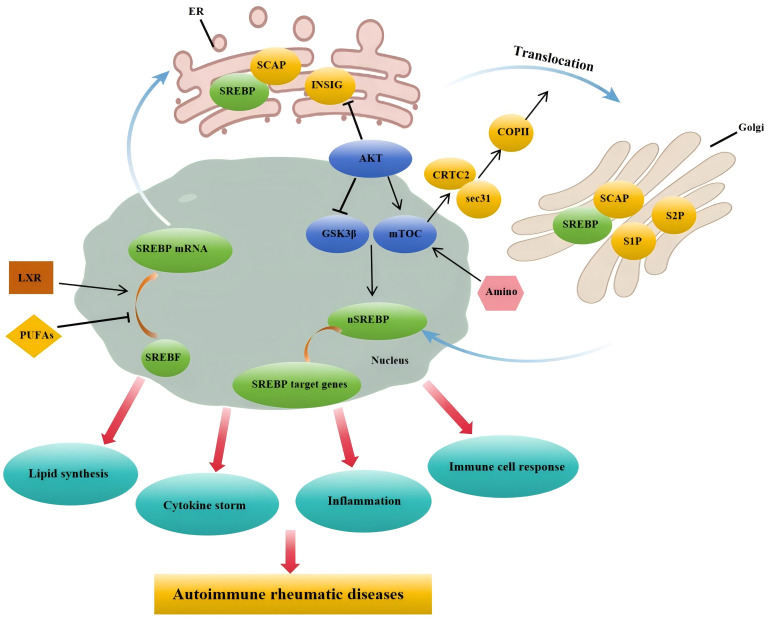

## 1 Introduction

Autoimmune rheumatic diseases represent a group of chronic disorders characterized by inflammation and autoimmunity, with the potential to affect any organ or system, leading to systemic damage. Current research indicates the involvement of various signaling pathways, including type I interferon pathways, immune cell pathways, immune metabolic pathways, complement, and coagulation, in the pathogenesis of these diseases. Adaptive immune cells, particularly B lymphocytes, play a significant role in the mechanisms underlying the development of autoimmune rheumatic diseases. B cells emerge as crucial contributors in many autoimmune disorders, such as RA, SLE, gout and multiple sclerosis (MS). In many of these conditions, the production of autoantibodies may constitute a primary pathogenic mechanism, highlighting the potential roles of B cell subsets and terminally differentiated antibody-producing plasma cells in autoimmunity (1). Innate immune cells mainly include macrophages, neutrophils, dendritic cells, natural killer cells, eosinophils, basophils, and other cell types, they circulate in the blood or reside in tissues, serving as the first line of defense against various pathogenic factors (2). Under the influence of relevant pathogenic factors, these cells produce cytokines or regulate the dynamic balance of tissue microenvironments through direct interactions with lymphocytes, participating in tissue damage and repair (3).

Inflammasomes, as crucial components of the innate immune response, play a vital role in the clearance of pathogens or damaged cells, providing a defense mechanism against pathogens and preventing pathological host damage. However, excessive activation of inflammasomes can also lead to autoimmune diseases, including RA, juvenile idiopathic arthritis (JIA), SLE and others (4–6). Inflammasomes and the interleukin-1 family of cytokines can promote the development of autoimmune diseases through the generation of adaptive immunity by T and B lymphocytes (4).

SREBPs were initially identified by Brown et al. as transcription factors that regulate the promoters of genes involved in cholesterol biosynthesis and the sterol regulatory pathway of the LDLR (7–9). Biological analysis underscores the significance of the SREBP pathway as a pivotal node in cell growth, metabolism, circadian rhythms, cellular stress, inflammation, and homeostasis (10). Recent research has indicated a correlation between SREBPs and various pathogenic processes, including endoplasmic reticulum (ER) stress, inflammation, cell apoptosis, and autophagy, with disease severity also being linked to SREBP levels (10–13). Although the SCAP-SREBP pathway plays a crucial role in cholesterol metabolism, its role in the immune system remains poorly understood. Recent studies have found that the activation of SREBP leads to inflammasome activation, induces macrophage inflammation, and triggers cytokine storms, thereby participating in related immune and inflammatory responses (14, 15). Additionally, research indicates a close association between the SREBP pathway-controlled cholesterol metabolism and adaptive immunity, including antibody and T follicular cell responses (16). Loss of SREBP in T cells severely impairs CD8 T cell activation (17). Moreover, the absence of SREBP signaling in B cells results in defective germinal center, memory B cell, and bone marrow plasma cell generation, preventing the generation of effective antibody responses (18).

Consequently, this article provides a comprehensive review of the SREBP’s upstream regulators, intracellular distribution, and identified biological functions. Furthermore, based on the established biological functions, we hypothesize that this protein may play a crucial role in the development of autoimmune rheumatic diseases such as SLE, RA and gout, and we present a series of prospects for future investigation in this field, to provide a novel target for the treatment of this type of disease.

## 2 Regulators of SREBPs

SREBPs are considered transcription factors that serve as primary regulators of a series of lipogenesis pathways, comprising three subtypes: SREBP-1a, SREBP-1c, and SREBP-2 (19). The activity of SREBPs relies on the SCAP (20). SCAP is a polytopic membrane protein residing in the endoplasmic reticulum. It binds to SREBP within the endoplasmic reticulum and transports it to the Golgi apparatus for proteolytic processing. Additionally, two other ER-resident membrane proteins, insulin-induced gene 1 protein (INSIG1) and INSIG2, interact with SCAP, causing the retention of the SREBP-SCAP complex in the ER membrane (21, 22). INSIG can enhance its own stability by binding to 25-hydroxycholesterol (produced in the ER). Once SREBPs leave the ER and enter the Golgi apparatus, they undergo a two-step proteolytic processing by two proteases: site-1 protease and site-2 protease (23). Cholesterol 25-hydroxylase, an enzyme responsive to type I interferons, increases the production of 25-hydroxycholesterol, which inactivates SREBPs and subsequently exerts anti-inflammatory effects by reducing the secretion of IL-1β (24, 25).

The liver X receptor (LXR) serves as a pivotal transcriptional regulator of cholesterol, fatty acid, and phospholipid metabolism (26). The promoter region of SREBP1c possesses an SRE element that facilitates its self-regulatory activation (27), LXRα and LXRβ are powerful activators of the SREBP1c promoter. When LXR agonists are administered, they stimulate the activation of both SREBP1c and fatty acid synthase (28). Consumption of polyunsaturated fatty acids (PUFAs) can reduce hepatic SREBP1c activity (29). The inhibition of SREBP1c by PUFAs occurs through several mechanisms: reduced transcription, increased mRNA decay, inhibition of proteolytic cleavage, and enhanced proteasomal degradation of nuclear SREBP1c (30).

ATF6 is a transcription factor anchored in the ER membrane, overexpression of exogenous, active ATF6 or its activation through glucose depletion has been observed to suppress the expression of SREBP target genes. Specifically, ATF6 is known to interact directly with SREBP2 through its leucine-zipper domain. This interaction recruits histone deacetylase 1, thereby inhibiting the transcriptional activity of SREBP2 (31). Amino acid levels play a crucial role in the activation and regulation of SREBP expression. Specifically, amino acids activate the mechanistic target of rapamycin complex 1 (mTORC1) within the lysosome. By modulating mTORC1 activity, amino acids ensure that cells can appropriately adjust to metabolic demands, impacting both anabolic and catabolic pathways (32, 33).

The PI3K-AKT-mTOR pathway has been identified as a major upstream signaling pathway regulating SREBP (34). AKT phosphorylates GSK3β, inhibiting its activity, which reduces SREBP degradation via the FBXW7-mediated ubiquitin proteasome system (35). AKT phosphorylates and inhibits INSIG2A, freeing the SREBP–SCAP complex for transport to the Golgi (36). AKT suppresses TSC, activating mTORC1, which phosphorylates and relocates lipin-1 to the nucleus, activating nuclear SREBP1 (37, 38). mTORC1 phosphorylates CREB-regulated transcription coactivator 2, promoting SREBP1 translocation from the ER to the Golgi by releasing inhibitory SEC31 and facilitating COPII vesicle formation (39).

## 3 Biological functions of SREBPs

### 3.1 The role of SREBPs in lipogenesis regulation

The SREBP1 gene gives rise to two isoforms through transcription from distinct promoters. SREBP1c is the predominant subtype expressed in most tissues, whereas SREBP1a exhibits high expression only in specific tissues and cells, such as intestinal epithelium, cardiac tissue, macrophages, and bone marrow dendritic cells (40). SREBP1c lacks 24 amino acid residues in the N-terminal transactivation domain of SREBP1a. This domain allows SREBP1a to bind tightly to CREB-binding protein, and as SREBP1c lacks these amino acids, its transcriptional activity is relatively lower (41, 42). In most cultured cells, SREBP1a (rather than SREBP1c) is the predominant isoform, possibly because SREBP1a can stimulate the expression of lipogenic and cholesterol-synthetic genes, thus providing components necessary for membrane lipid synthesis (43, 44).

A series of animal studies employing transgenic and knockout mice for each SREBP gene and subtype indicate that SREBP1c primarily regulates the expression of fat synthesis genes, while SREBP2 controls genes related to cholesterol metabolism (20, 41, 43). Physiologically, SREBP1a robustly activates total fat synthesis in rapidly growing cells, while SREBP1c plays a role in the nutritional regulation of fatty acids and triglycerides in fat-producing organs, such as the liver. In contrast, SREBP2 has a regulatory role in all tissues (20). This functional specificity is more evident *in vivo* than *in vitro*, but isoforms exhibit functional overlap when overexpressed. The partial specificity of SREBP subtypes for different target genes is explained by their unique binding dynamics with cholesterol synthesis genes, mainly SRE elements, as well as SREs and enhancer boxes in fat genes, and auxiliary factors such as SP1 and NFY (45). SREBP1a and SREBP2, but not SREBP1c, associate as coactivators with CBP and P300 (also known as EP300) to recruit the mediator complex (42). The SREBP family is also acetylated and stabilized by CBP and P300 (42, 46, 47).

### 3.2 SREBPs can elicit cytokine storms

Cytokine storms refer to the rapid and excessive release of pro-inflammatory cytokines by immune cells, which can lead to a hyperinflammatory response and contribute to various pathological conditions. The mechanisms through which SREBPs induce cytokine storms and their precise impact on immune regulation are areas of ongoing research. SREBP2 activation induces an inflammatory response and exacerbates inflammatory damage. Firstly, existing research suggests that SREBP2 can regulate the inflammatory phenotype by modulating cholesterol homeostasis. Increased cholesterol synthesis is involved in various immune pathways, such as interferon response, inflammasome activation, and innate immunity (14, 48, 49). Additionally, perturbation of cellular cholesterol may alter membrane dynamics and impact cell signal transduction (50). Secondly, SREBP2 can interact with several pro-inflammatory mediators and promote their transcription, such as IL1β, IL8, NLRP3, and NOX2 (51–54).

SREBP-2 serves as a transcription factor for lipid synthesis. However, it has been observed that COVID-19 patients maintain lower cholesterol levels, even though the expression levels of SREBP-2 in their plasma increase. A seminal discovery by Wonhwa (15) identified an elevated C-terminal segment of SREBP-2, termed SREBP-2C, in the blood of COVID-19 patients. This heightened level of SREBP-2C correlates closely with the excessive inflammation observed in the lung tissues of COVID-19 patients, inducing an upregulation of inflammatory responses that can lead to cytokine storms. Clinical studies have further revealed that ICU patients with elevated plasma levels of SREBP-2C exhibit more severe lung inflammation in CT images compared to non-ICU patients with lower SREBP-2C levels. Consequently, SREBP-2 can serve as a diagnostic marker for the severity of COVID-19 in critically ill patients and as a therapeutic target for preventing cytokine storms and lung damage. Additionally, in an infectious disease mouse model, the inhibition of SREBP-2 and NF-κB attenuated cytokine storms induced by viral infection and prevented lung injury. These findings underscore the potential clinical significance of SREBP-2 in assessing COVID-19 severity and its role in the prevention of cytokine storms and lung injury, offering novel prospects for the diagnosis and treatment of COVID-19.

### 3.3 SREBPs can promote macrophage inflammation

The inflammasome is a multiprotein complex formed in the cytoplasm following exposure to various stimuli from pathogenic sources. Its activation depends on sensor proteins recognizing ligands, subsequently recruiting adaptor protein ASC (55). This leads to ASC oligomerization and the recruitment and activation of caspase-1, an enzyme responsible for processing Pro-IL-1β into mature IL-1β. Studies indicate that conditioned media from BMDMs transduced with Hmgcr or Dhcr24 enhances their ability to stimulate T cells for IL-17A production (56). Furthermore, researchers have demonstrated that cholesterol-treated BMDM significantly promote IL-1β production. Inhibition of SREBP expression can suppress IL-1β-induced macrophage inflammation (14).

In macrophages, studies have revealed that the SCAP/SREBP2 shuttle complex directly interacts with the NLRP3 inflammasome, regulating inflammasome activation by translocating from the endoplasmic reticulum to the Golgi apparatus (57). Another set of research indicates that SREBP2 is highly activated in macrophages treated with TNFα, and nuclear SREBP2 binds to target genes involved in inflammation and interferon responses, promoting an M1-like inflammatory state (51). Moreover, several studies suggest that cellular cholesterol levels control immune phenotypes. The type I interferon (IFN) signal in macrophages reduces cholesterol synthesis, allowing the activation of STING on the ER to enhance IFN signaling (49). Furthermore, research has shown that restoring cholesterol biosynthesis in macrophages promotes inflammation (14, 50).

Additionally, inflammatory factors upregulate SCAP expression, facilitating the translocation of the SCAP/SREBP2 complex from the endoplasmic reticulum to the Golgi apparatus. This disrupts intracellular cholesterol homeostasis and contributes to atherosclerosis and non-alcoholic fatty liver disease (58, 59). We have also discovered that crosstalk between SCAP/SREBP and the TLR4-MyD88-NF-κB inflammation pathway mediates foam cell formation in atherosclerosis (60). Moreover, SCAP overexpression promotes the translocation of SCAP and NLRP3 inflammasomes to the Golgi apparatus, increasing the activation of the NLRP3 inflammasome pathway and thereby expediting atherosclerosis (61, 62). These findings highlight SCAP as a crucial molecular link between lipid metabolism and inflammation (63). The STING/TBK1 pathway, a classical innate immune signaling pathway, has recently been shown to play a critical role in the inflammatory response of metabolic diseases. SCAPs activation of the STING-NF-κB signaling pathway has been implicated in the pathogenesis of macrophage inflammation and lean non-alcoholic fatty liver disease (64).

### 3.4 SREBPs can regulate cellular energy metabolism to promote the proliferation of B cells and the production of autoantibodies

Recent research indicates that the SREBP pathway, governing sterol metabolism, is closely associated with adaptive immunity, including antibody and T follicular cell responses (16). Depletion of SREBP in T cells severely impedes CD8 T cell activation (17). In addition, recent research has revealed a connection between B-cell activation and lipid metabolism reprogramming (18). Activation of TLR4, CD40, and BCR signaling pathways significantly upregulates the expression of most genes involved in cholesterol biosynthesis in B cells. Notably, the lack of SCAP severely inhibits the regulation of these cholesterol biosynthesis-related genes, including those encoding key enzymes such as Hmgcr, Hmgcs1, Sqle, Dhcr24, and lipid synthesis-related genes Acsl3 and Acsl4. Pathway analysis further indicates that the lipid biosynthesis pathway is one of the highly impacted pathways associated with SCAP deficiency in B cells.

Studies have demonstrated that B cells undergo 2-5 rounds of division after stimulation with different TLRs or CD40, while SCAP-deficient B cells not only fail to undergo division but are also blocked from entering the S phase. This suggests that the SREBP signal is a key factor in mitogen-induced B-cell division and promotes the B-cell cycle process. Furthermore, SCAP deficiency leads to a sharp reduction in CD138 and YFP-positive GC-derived plasma cells, resulting in a significant decrease in antigen-specific IgG titers and reduced affinity maturation. These findings highlight the critical role of cholesterol synthesis-mediated SREBP signaling in maintaining lipid homeostasis, cell cycle progression, and plasma cell differentiation. Loss of SREBP signaling in B cells impairs the generation of germinal center, memory B cells, and bone marrow plasma cells, preventing the effective production of antibody responses (18).

### 3.5 SREBPs can facilitates cancer development

Dysregulated cellular lipid metabolism, driven by the SREBP pathway, is a prominent feature of cancer cells (65). Oncogenic growth signals render cells reliant on *de novo* lipogenesis, which encompasses extensive fatty acid synthesis from glucose and glutamine to meet the bioenergetic and biosynthetic demands of rapidly proliferating tumor cells (66). Insulin-mediated signals through the PI3K-AKT-mTORC1-SREBP axis play a pivotal role in regulating lipid synthesis in response to nutritional fluctuations. This signaling pathway also serves as an established survival route, activated structurally in many cancer types, exerting pronounced roles in growth, malignancy, anti-apoptosis, drug resistance, and metastasis (67). Numerous oncogenic signaling molecules, including P53, PTEN, PI3K, and KRAS, converge upon the PI3K-AKT-mTOR pathway to activate protein and lipid biosynthesis in cancer cells, satisfying the demand for lipids in cell growth. Even in conditions of low oxygen and high acidity, SREBP1a mediates the metabolic flux from enhanced glycolysis to lipid synthesis via PI3K-AKT signaling, upregulating the LDLR to facilitate cholesterol uptake. SREBP1a is highly expressed in cancer cells and exhibits robust promoter activity in actively proliferating cancer cells (68–71).

The mevalonate pathway is upregulated in many cancers, including liver cancer, possibly due to mutations in sterol-related genes, such as SREBP2 and SCAP, resulting from p53 mutations (71). Additionally, genome-wide expression analysis identified mutations in sterol gene promoters for p53 and SREBP in human breast tumors. These mutations disrupted breast tissue architecture through the mevalonate pathway (69–71). The tumor suppressor retinoblastoma protein and GTPase NRAS interact in an anticancer senescence pathway. retinoblastoma protein loss activates SREBPs and enhances geranylgeranylation in a reverse E2F-dependent manner, leading to NRAS activation, subsequent induction of DNA damage response, and p130-dependent cell senescence (72), thus promoting retinoblastoma development.

## 4 Prospects of SREBPs in rheumatic immune diseases

### 4.1 SREBP and systemic lupus erythematosus

SLE is a chronic autoimmune disease characterized by genetic, endocrine, environmental, and their interaction-induced autoantibody production, immune complex deposition, abnormal activation of various immune cells (such as T and B lymphocytes and granulocytes), and tissue damage in organs like the kidneys, skin, heart, and lungs (73–75). The exact etiology and pathogenesis of SLE remain incompletely understood (76). In China, the incidence of SLE is approximately 70 per 100,000, and it shows an increasing trend year by year (75). SLE has a complex pathogenesis closely related to genetics, immune dysregulation, viral infections, and environmental factors (77, 78). Recent research on the pathogenesis of SLE has provided a wealth of information (79–90). Among the multitude of mechanisms contributing to SLE, immune dysregulation stands out as one of the primary drivers of disease. Immune cells play a pivotal role in the initiation and progression of SLE’s autoimmune response. In SLE, immune cells exhibit overexpression of autoimmunity, imbalanced cytokine production, and increased apoptosis, all of which have a significant impact on the pathogenesis of SLE (91).

**Figure 1:**
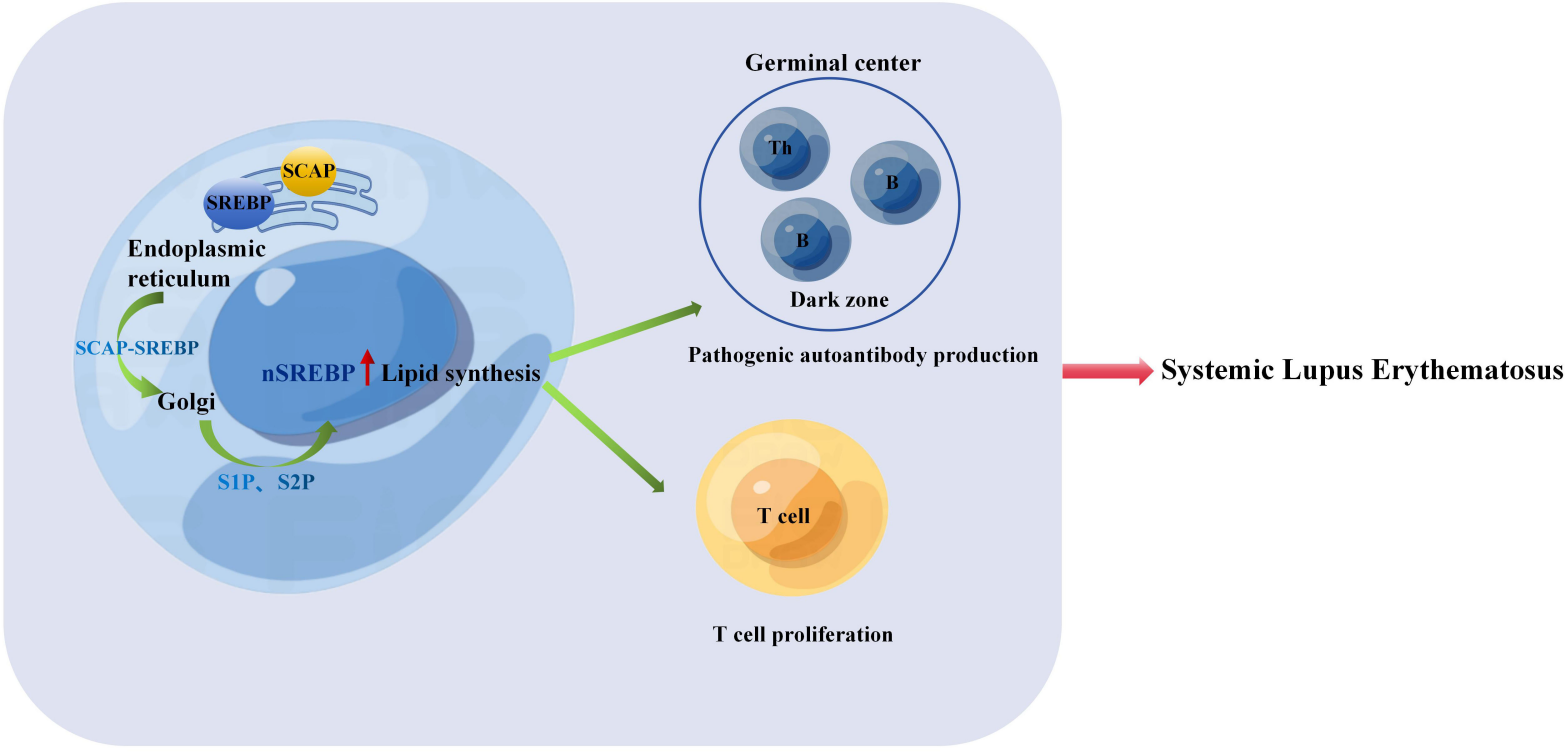
The SREBP-SCAP complex is transported from the endoplasmic reticulum to the Golgi apparatus and into the nucleus. Thus, lipid synthesis increases, promoting T cell proliferation and the process of B cell cycle is accelerated, B cells are activated and proliferated to form GC, and then B cells complete Ig affinity maturation and class conversion in GC, which leads to excessive expression of pathogenic autoantibodies, autoantibodies combine with antigens to form immune complexes, and then lead to the pathogenesis of SLE.

Lipids are crucial constituents of cell membranes, and changes in their composition and content can influence the normal function of cells. For instance, alterations in the composition, distribution, and dynamics of lipid rafts (microdomains primarily composed of cholesterol and sphingolipids) on T cells accelerate their activation in SLE patients, exacerbating the condition (92). Anomalies in lipid raft expression on T cells in SLE may lead to abnormal activation and signaling pathways, resulting in the production of aberrantly expressed cytokines and assisting in the abnormal response of B lymphocytes, leading to the production of autoantibodies and the development of SLE (93). Additionally, the cellular microenvironment plays a significant role in influencing cellular lipid metabolism, further contributing to cellular dysfunction (94). Research indicates that cholesterol buildup is essential for T cell proliferation and their response to antigen interactions. When cholesterol synthesis is blocked due to SCAP deficiency, T cell proliferation is entirely halted (17, 95). On the other hand, if cholesterol cannot be esterified because of Acat1 deficiency, there is an increase in plasma membrane cholesterol accumulation, which enhances T cell proliferation (96). Likewise (97), the lack of Abcg1-mediated cholesterol efflux results in increased plasma membrane cholesterol and further promotes T cell proliferation (**Figure 1**).

Research (98) has indicated a close relationship between IFN-γ expression and the severity of SLE in both human and murine models of the disease, the increase in nuclear form of SREBP (nSREBP) leads to cholesterol accumulation, which promotes the expression of IFN-γ (99). Spontaneously developed germinal centers containing autoreactive B cells that produce pathogenic autoantibodies play a role in promoting autoimmune responses and driving the development of systemic lupus erythematosus. Studies have demonstrated that IFN-γ-STAT1 signaling controls the formation of GCs by promoting the expression of T-bet in B cells and the production of IFN-γ (100). As previously discussed, the loss of nSREBP signaling in cells can lead to defects in cell cycle progression and metabolic reprogramming, thereby impeding the generation of germinal centers, memory B cells, and plasma cells required for an effective antibody response (18). We speculate that increased expression of nSREBP in B cells accelerates B cell cycle progression, promotes B cell activation and proliferation, leading to the formation of germinal centers. Subsequently, B cells complete Ig affinity maturation and class switching in germinal centers, resulting in excessive production of pathogenic autoantibodies. The binding of these autoantibodies to antigens forms immune complexes, ultimately contributing to the pathogenesis of SLE (**Figure 1**).

**Figure 2:**
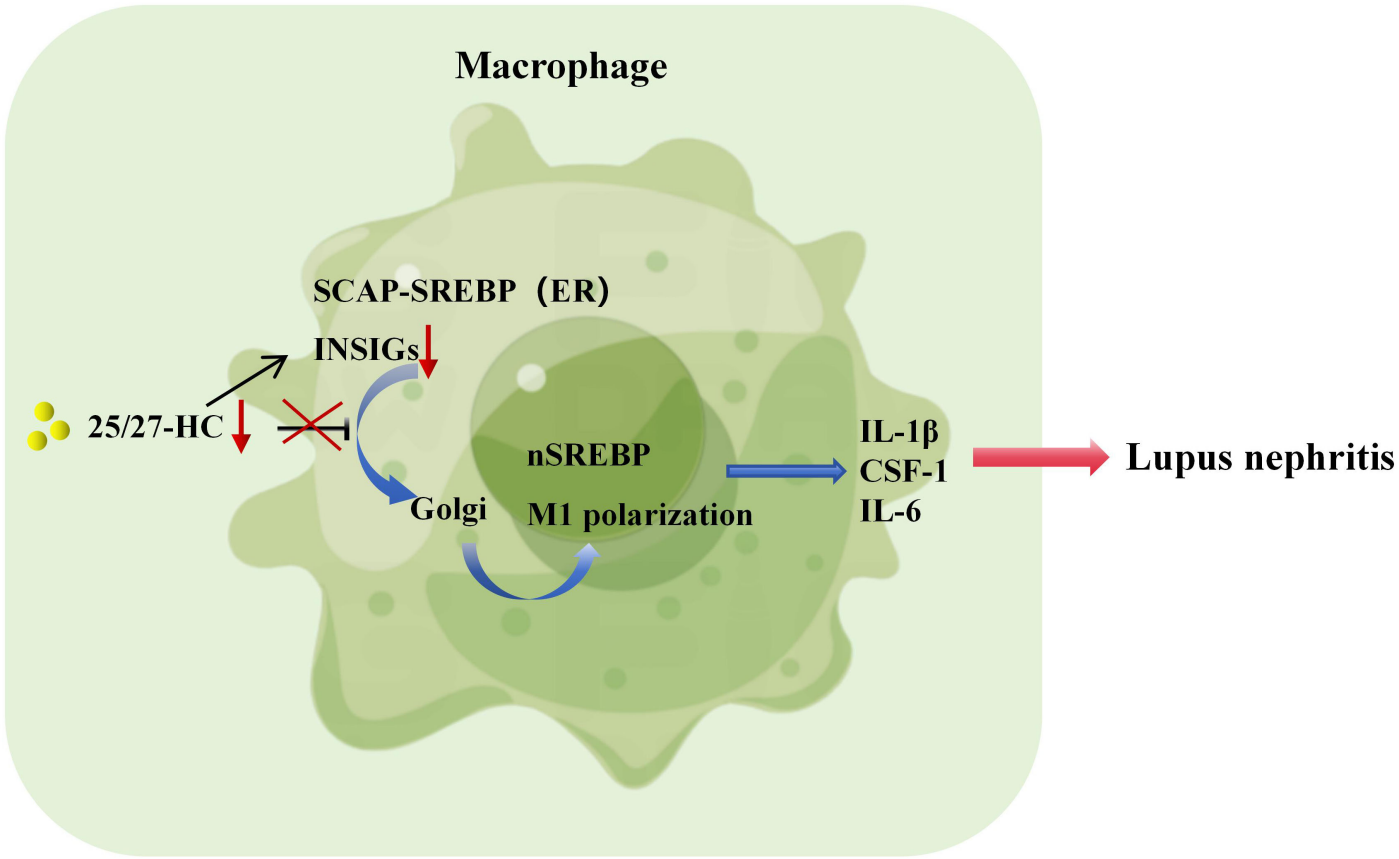
SREBP-regulated macrophage inflammation promotes the pathogenesis of lupus nephritis. If the expression of 25/27-HC is too low and INSIGs cannot inhibit the transport of SREBP-SCAP complex to Golgi, it will lead to the overexpression of SREBP, produce a large amount of IL-1β, IL-6 and CSF-1, promote the expansion of M1 macrophages and accelerate the pathogenesis of lupus nephritis.

Macrophages can be polarized into inflammatory (M1) and anti-inflammatory (M2) phenotypes through various stimuli, such as IFN-γ and LPS for M1 polarization, or IL-4 for M2 polarization, playing crucial roles in immune regulation (101). Studies have shown that M1 macrophages in patients with SLE can regulate the activation status of T and B lymphocytes, thereby influencing disease activity (102). In MRL/lpr mice, transient ischemic renal injury upregulates CSF-1 expression in renal tubular epithelial cells, leading to increased release of CSF-1 and subsequent expansion of M1 macrophages, accelerating the onset of lupus nephritis (103). Moreover, recent research has revealed that human and murine macrophages, when stimulated by IgG immune complexes, undergo metabolic reprogramming dependent on mTOR and HIF-1α, resulting in the production of pro-inflammatory cytokine IL-1β, thereby promoting lupus nephritis (104). In the presence of cholesterol and oxygenated sterols, including 25/27-HC, the SREBP-SCAP complex remains sequestered within the endoplasmic reticulum along with INSIGs. Studies have indicated (15) that overexpression of nSREBP leads to a cytokine storm, while inhibiting nSREBP expression can suppress the production of inflammatory cytokines such as IL-1β and TNF-α, dampening macrophage inflammatory responses. Consequently, it is hypothesized that when 25/27-HC expression is reduced, preventing INSIGs from inhibiting the translocation of the SREBP-SCAP complex to the Golgi apparatus (**Figure 1**), excessive SREBP expression ensues, resulting in the overproduction of IL-1β, IL-6, and CSF-1, which promotes the expansion of M1 macrophages and accelerates the onset of lupus nephritis (**Figure 2**).

All of these findings collectively suggest that SREBPs may play a crucial role in the pathogenesis of SLE. Investigating the mechanistic involvement of SREBPs in SLE could offer a novel therapeutic target for the treatment of SLE.

### 4.2 SREBP and Rheumatoid Arthritis

RA is an autoimmune disease characterized by progressive synovial inflammation in multiple joints, resulting in widespread symmetrical joint swelling, pain, bone destruction, joint deformities, and potential involvement of connective tissues such as the heart, lungs, and eyes. It is considered a severe and challenging-to-treat disease (105). Epidemiological studies have indicated a global distribution of RA, with an average prevalence ranging from 0.5% to 1.5%. In China, the prevalence of RA in adults is approximately 0.4% and shows an increasing trend (106). The etiology and pathogenesis of RA remain inconclusive, but it is generally accepted that RA is an inflammatory disease triggered by external factors on a genetic susceptibility background. RA patients exhibit innate immune and adaptive immune response abnormalities, characterized by an imbalance in Th1/Th2 responses, an increased number of Th17 cells, and compromised functions of T and B lymphocytes that have immunosuppressive effects. These abnormalities lead to the production of a multitude of pro-inflammatory cytokines, including IFN-γ, TNF-α, IL-1β, IL-6, and IL-17 (107–109), which participate in a positive feedback loop, promoting the continuous progression of inflammation. Ultimately, this results in the infiltration of inflammatory cells into the synovium, cartilage and bone destruction, joint deformities, and peripheral tissue damage in RA patients. SREBP-2 promotes targets of the methoxyerate pathway and interferon-responsive genes in TNF-activated macrophages (51). Recent studies have shown that endotoxin-mediated IL-1β production is partially dependent on SREBP-1a. Caspase-11 interacts with the S1P-SREBP pathway, activating SREBP-1a, which subsequently drives downstream inflammatory responses (40, 110). Given the characteristics of SREBP overexpression promoting the release of inflammatory factors, we speculate that SREBP exacerbates the continuous progression of inflammation, leading to synovial infiltration of inflammatory cells, cartilage and bone destruction, joint deformities, and peripheral tissue damage in RA patients.

The JAK-STAT signaling pathway plays a significant role in the pathogenesis of RA (111–114). JAK2 activates downstream genes by phosphorylating STAT3 or STAT5, leading to the release of pro-inflammatory signals, such as IL-6, IFN-γ, IL-12, and other inflammatory factors (115–118). Researches have shown that blocking the JAK2-STAT3 pathway by activating AMPK can exert an anti-inflammatory effect. AMPK phosphorylates JAK2 at Ser515 and Ser518, inhibiting JAK2-mediated STAT3 activation (119, 120). Furthermore (121), study has shown that the activation of AMPK can suppress the expression of SREBP, while AMPK expression is inhibited in RA. Therefore, we speculate that SREBP may also play a role in the pathogenesis of RA.

**Figure 3:**
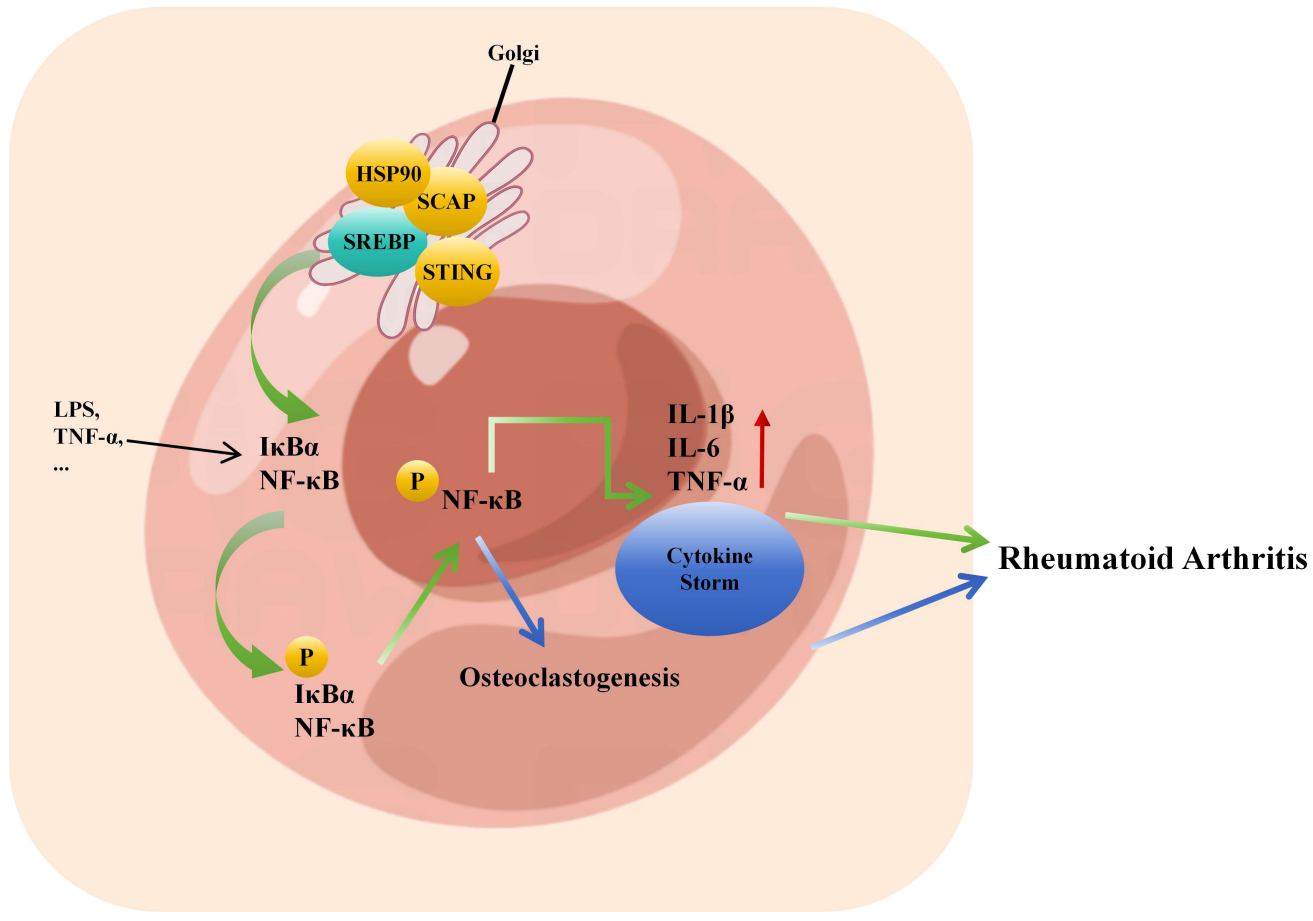
Inflammatory factor storm caused by crosstalk between SREBP and NF-κB promotes the pathogenesis of rheumatoid arthritis. SCAP/SREBP/STING/TBK1 can activate the transcriptional expression of downstream inflammatory factors such as IL-1β, IL-6, IL-17, TNF-α by activating NF-κB phosphorylation, thus reactivating NF-κB, resulting in further amplification of the initial inflammatory signal, the formation of inflammatory factor storm, leading to joint inflammation to accelerate the pathogenesis of RA. HSP90β promotes osteoclast formation by activating SREBP2 to increase cholesterol biosynthesis and NF-κB signal, which leads to the pathogenesis of RA.

NF-κB plays an essential role in joint destruction. When cells are stimulated, IκB are phosphorylated by IκB kinase, leading to the release of NF-κB. NF-κB enters the cell nucleus and, through a series of reactions, initiates the transcription and expression of downstream inflammatory factors such as IL-1β, IL-6, IL-12, IL-17, TNF-α, and others (122). This, in turn, activates NF-κB, causing an amplification of the initial inflammatory signal, resulting in a cascade reaction that sustains the development of the inflammatory response and structural damage. Clinical studies have found significantly elevated expression levels and activity of NF-κB in the serum and lymphatic endothelial cells of RA patients. Animal experiments have also confirmed that inhibiting the NF-κB pathway can significantly improve the degree of toe swelling and inflammatory responses in a mouse model of RA (123). Studies have found that SCAP and SREBP1 form a super complex with IkBα, bringing NF-kB close to the endoplasmic reticulum. Upon endotoxin stimulation, SCAP escorts this complex to the Golgi apparatus, where SREBP1 is cleaved by S1P/S2P, releasing IkBα for phosphorylation and activating subsequent inflammatory responses (124, 125). Additionally, the SCAP/SREBP/STING/TBK1 pathway can activate NF-κB in metabolic diseases and promote the expression of related inflammatory factors (64). HSP90 is a new regulatory factor of SREBP and can bind to the SREBP-SCAP complex in the endoplasmic reticulum and Golgi apparatus, stabilizing it. The inhibition of HSP90 results in the dependence of the complex on proteasomal degradation (126). HSP90β activates SREBP2, increasing cholesterol biosynthesis and NF-κB signaling to promote osteoclastogenesis (127). Inhibiting SREBP and subsequently suppressing NF-κB-related inflammatory responses may alleviate inflammation, inhibit bone destruction, reduce disability, and achieve disease remission in RA patients (**Figure 3**). This hypothesis merits further investigation.

### 4.3 SREBP and gout

Gout is an inflammatory disease characterized by the massive deposition of urate crystals in joints and surrounding tissues, resulting from disturbances in purine metabolism and/or abnormal uric acid excretion (128). It has become the second most prevalent metabolic disease in China, following diabetes (129). Recurrent gout attacks not only cause severe pain in affected joints but can also lead to joint damage, dislocation, deformities, and even joint disability or amputation. Furthermore, the extensive deposition of urate crystals in the kidneys can lead to renal damage and even acute kidney failure, which can be fatal (130). Currently, commonly used urate-lowering drugs such as febuxostat, while effective to some extent, have the potential for severe toxic side effects with long-term use, low patient tolerance, and a high disease recurrence rate, significantly impacting patient compliance (131).

Modern medicine recognizes that MSU crystals can strongly stimulate Toll-like receptors and the NLRP3 inflammasome, leading to the activation of the innate immune response, primarily involving macrophages and neutrophils. This eventually results in the release of IL-1β and other pro-inflammatory factors, triggering gout attacks (132, 133). Macrophages play a frontline role in the immune response, with different subtypes classified based on the cytokines they secrete and the cell surface adhesion molecules. Among these subtypes, M1 macrophages, when activated, promote the assembly of the NLRP3 inflammasome, leading to the release of high pro-inflammatory cytokines such as TNF-α and IL-1β, along with promoting the recruitment of neutrophils from peripheral blood to the inflammatory site, subsequently releasing pro-inflammatory cytokines and causing sustained inflammation. In contrast, M2 macrophages can suppress MSU crystal-induced inflammation and inhibit caspase-1 activation and IL-1β production (134). In M1 pro-inflammatory macrophages, the TCA cycle is disrupted, whereas M2 macrophages possess a complete TCA cycle and mainly rely on OXPHOS (135). The overexpression of SREBP1a promotes the repositioning of the NLRP3 inflammasome to the Golgi apparatus, thereby enhancing the activation of the NLRP3 inflammasome pathway (61, 62). SREBP1a also directly activates the transcription of NLRP genes and caspase-1, mediating the secretion of IL-1β by macrophages. This indicates an important function of macrophage SREBP1a, associating lipid generation and/or lipid toxicity with innate immune responses (40). MIR-33 is a microRNA involved in SREBP signaling, and is also involved in the production of pro-inflammatory and anti-inflammatory genes in M1 and M2 macrophages, respectively (136). In addition, SREBP and miR-33 inhibit cholesterol efflux through the ATP-binding cassette transporter A1 in macrophages (137). Loss of SCAP caused changes in cholesterol metabolism and induced proinflammatory M1 polarization in adipose tissue macrophages (125, 138).

**Figure 4:**
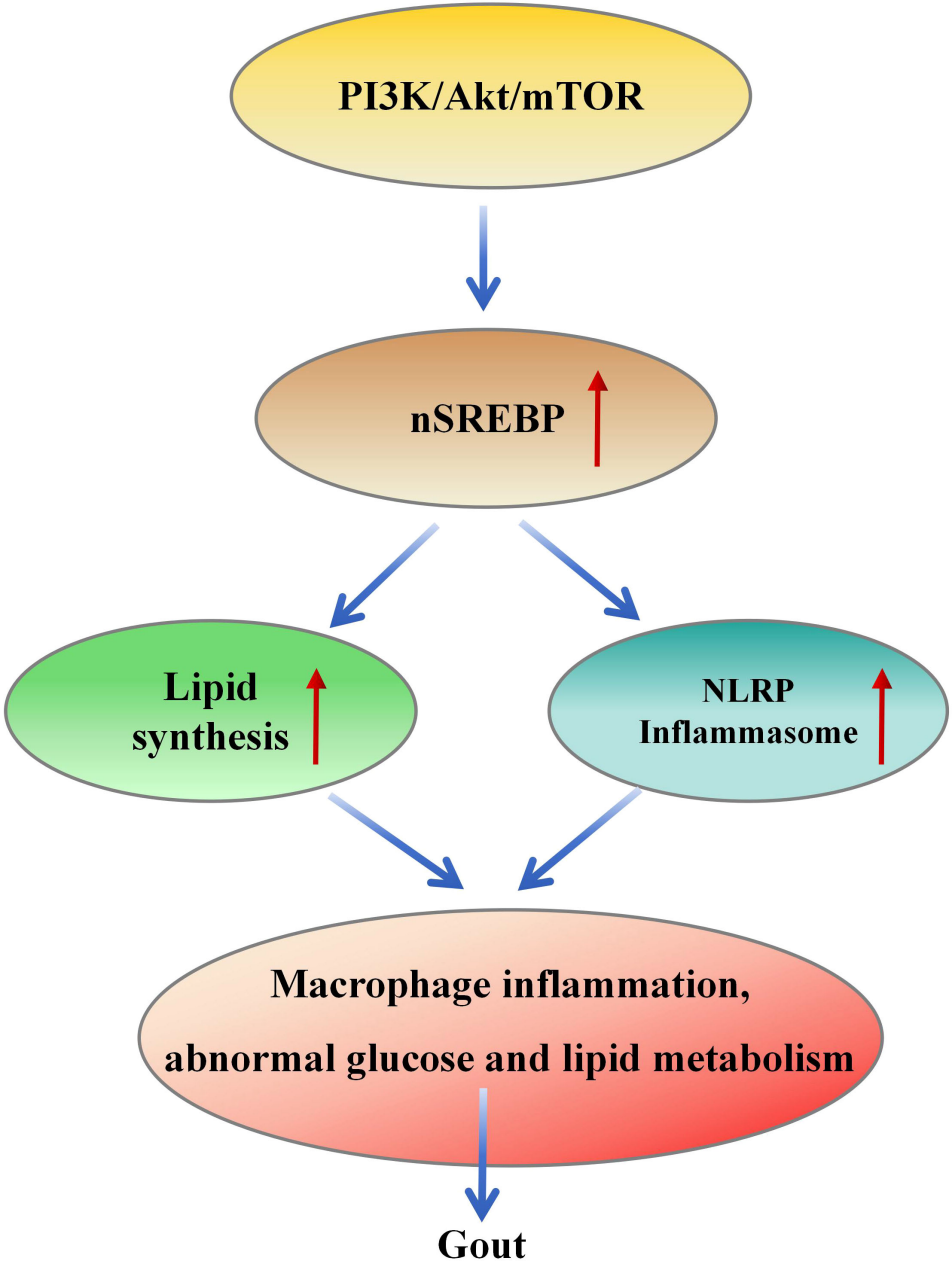
PI3K/Akt/mTOR/SREBP signal transduction leads to the pathogenesis of gout. PI3K/Akt/mTOR signal activates SREBP, resulting in increased fat production and the formation of inflammatory bodies, which further leads to macrophage inflammation and abnormal glucose and lipid metabolism, resulting in the pathogenesis of gout.

Macrophage polarization is a complex process influenced by the interaction of various intracellular signaling molecules and pathways. It holds crucial significance in inflammation, metabolic diseases, autoimmune disorders, and other conditions. Among these factors, the regulation of the PI3K/Akt/mTOR signaling pathway plays a central role in controlling macrophage polarization (139). Furthermore, SREBP1 mediates metabolic flux from enhanced glycolysis to lipid generation via the PI3K-AKT pathway and upregulates LDLR, promoting cholesterol uptake (68–71). These factors can lead to macrophage inflammation, glucose and lipid metabolic abnormalities, ultimately contributing to the pathogenesis of gout (**Figure 4**).

While macrophages and neutrophils are considered the primary immune cells involved in gout pathogenesis (140, 141), recent researches have highlighted the role of T cell subsets in gout (142, 143). Targeting pro-inflammatory T cell subsets or their associated cytokines can improve MSU crystal-induced arthritis in mice. The gut microbiota and its metabolites play a significant role in maintaining intestinal homeostasis (144, 145) and regulating T cell differentiation (146), which are crucial in the development of autoimmune disorders and inflammatory diseases. A previous study indicated that the loss of SCAP in T cells severely impairs CD8 T cell activation (17). Due to the limited research on SREBP in gout, future investigations targeting this pathway may provide new strategies for gout treatment.

## 5 Conclusions

SREBP has emerged as a significant discovery in medical research in recent years, research on SREBP has primarily focused on the field of obesity and cancer. However, the functions of SREBP in lipid metabolism, cell growth, and inflammation are also closely related to the pathogenesis of rheumatic and immune diseases. Therefore, investigating the expression characteristics and signaling pathways of SREBP in patients with rheumatic immune diseases can provide deeper insights into the pathogenesis of these conditions. This, in turn, holds substantial significance for the identification of early biomarkers and the development of precise personalized treatment approaches.

## Glossary

AMPK: adenine mononucleotide activated protein kinase
AKT: protein kinase B
BMDM: bone marrow-derived macrophage
CREB1: cAMP responsive element binding protein1
CSF-1: colony-stimulating factor-1
GC: germinal center
GTP: guanosine triphosphate
HIF-1α: hypoxia-inducible factor-1α
HSP90: heat shock protein 90
IFN-γ: interferon-γ
IL-1β: interleukin-1β
IL-4: interleukin-4
INSIGs: insulin -induced gene protein
IκB: NF-κB inhibitor proteins
JAK-STAT: janus kinase-signal transducer and activator of transcription
KRAS: kirsten rat sarcoma viral oncogene homolog
LECs: lymphatic endothelial cells
LDLR: low density lipoprotein receptor
LPS: lipopolysaccharide
M1: macrophages can polarize to inflammatory phenotype
mTOR: mammalian target of rapamycin
MyD88: myeloiddifferentiation factor88
MSU: monosodiumuratecrystals
NF-κB: nuclear factor-κB
NLRP3: nucleotide-binding oligomerization domain, leucine- rich repeat and pyrin domain-containing 3
NRAS: neuroblastoma RAS viral oncogene homolog
OXPHOS: oxidative phosphorylation
PI3K: phosphatidylinositol 3-kinase
PTEN: phosphatase and tensin homolog
RA: rheumatoid arthritis
SCAP: SREBP cleavage activating protein
SLE: systemic lupus erythematosus
SREBPs: sterol regulatory element binding proteins
STING: stimulator of interferon genes
TAC: tricarboxylic acid
TBK1: TANK-binding kinase 1
TLR4: toll-like receptor 4
25/27-HC: 25-hydroxycholesterol and 27-hydroxycholesterol
